# Co-circulation of multiple reassortant H6 subtype avian influenza viruses in wild birds in eastern China, 2016–2017

**DOI:** 10.1186/s12985-020-01331-z

**Published:** 2020-04-29

**Authors:** Chuanxia Hu, Xiaofang Li, Caihui Zhu, Feng Zhou, Wangjun Tang, Di Wu, Zhihui Li, Lichen Zhou, Jing Liu, Xiaoman Wei, Jie Cui, Tianhou Wang, Guimei He

**Affiliations:** 1 School of Life Sciences, East China Normal University, Shanghai, China; 2 Shanghai Zoo, Shanghai, China; 3 Jinshan Forest Working-Station, Shanghai, China; 4 Shanghai Wildlife Conservation and Management Center, Shanghai, China; 5 Institute of Eco-Chongming (IEC), East China Normal University, Shanghai, China; 6 Key Laboratory of Special Pathogens and Biosafety, Center for Emerging Infectious Diseases, Wuhan Institute of Virology, Chinese Academy of Sciences, Wuhan, China; 7 Unit of Pathogen Bioinformatics, CAS Key Laboratory of Molecular Virology and Immunology, Institut Pasteur of Shanghai, Chinese Academy of Sciences, Shanghai, 200031 China; 8 University of Chinese Academy of Sciences, Beijing, China

**Keywords:** H6 subtype avian influenza virus, Novel, Reassortant, Wild birds, Shanghai, Eastern China

## Abstract

**Background:**

H6 subtype influenza viruses were prevalent in domestic poultry and wild birds, which also could pose potential threat to humans. However, little is known about the prevalence of H6 subtype viruses in wild birds in eastern China, a crucial stopover or wintering site for migratory wild birds along the East Asian-Australasian Flyway.

**Methods:**

During the routine surveillance in 2016–2017, H6 subtype AIVs positive samples were identified, and the representative strains were selected for further sequence and phylogenetic analysis and the pathogenicity in mice were evaluated.

**Results:**

Among the 30 H6 positive samples, there were at least four subtypes H6N1, H6N2, H6N5 and H6N8 co-circulated in Shanghai, China. Genetic analysis showed the 8 representative isolates shared homology with different AIV sub-lineages isolated from domestic ducks or wild birds in different countries along the East Asian-Australasian flyways, and were classified into 7 new genotypes. The pathogenicity to mice showed that these H6 viruses could replicate efficiently in the lungs without prior adaptation, but could not cause mice death.

**Conclusions:**

Eight novel strains belonged to H6N1, H6N2, H6N5 and H6N8 subtypes were isolated. Phylogenetic analyses revealed multiple origins of internal genes indicative of robust reassortment events and frequent wild birds-poultry interaction encouraging the evolution and emergence of new genotypes. The pathogenicity to mammals should be closely monitored to prevent the emergence of novel pandemic viruses.

## Background

The H6 subtype avian influenza virus (AIV) was first isolated from a turkey in Massachusetts, United States in 1965 [1], and has been subsequently isolated from migratory waterfowl, as well as domestic aquatic and terrestrial avian species throughout the worldwild [2–4]. Surveillance suggested that H6 subtype AIVs have been one of the most commonly recognized subtypes, and several different subtypes of H6 AIVs co-circulate in domestic chickens and ducks in southern China [5, 6], which provides the opportunity for gene exchange between these viruses [7, 8]. The H6 subtypes might cross the species barrier and infected humans without prior adaption. During the outbreak of human H5N1 in Hong Kong in 1997, an H6N1 avian influenza virus in a live poultry market was isolated. Genetic characterization of this H6N1 virus revealed that its seven gene segments were closely related to human influenza virus A/HongKong/156/97(H5N1) [9, 10]. Moreover, human serological surveillance showed that serum that was antibody positive for H6 subtype AIVs could be found [11]. In 2013, the first human case of H6 subtype AIVs infection was reported in Taiwan, suggesting that the H6 subtype AIVs can cross the species barrier and infect humans [12]. A study also showed that 34% of the H6 subtype AIVs isolated from live poultry markets in southern China were able to bind human type receptors and some of them possessed the ability to transmit efficiently to contact animals [13]. These events all indicated that the H6 subtype AIVs are a potential threat to human health.

Shanghai, located at the Yangtze River estuary on the eastern coast of China, is a crucial stopover or wintering site for migratory wild birds along the East Asian-Australasian Flyway. As the natural host of AIVs, wild birds play a vital role in influenza virus’ reassortment and transmission. An extensive epidemiological surveillance study from 2002 to 2010 revealed that five subtypes of H6 AIVs co-circulated in poultry in eastern China, and that they were underwent frequent reassortment [14]. However, viruses isolated from wild birds were seldomly reported. In this study, we investigated several novel H6 subtype AIVs isolated from wild birds, in Shanghai, eastern China, during 2016–2017. Our research fills a major gap that wild birds do as the host of H6 subtype AIVs and interaction with poultry frequently occurred in the past.

## Materials and methods

### Sample

**Fig. 1:**
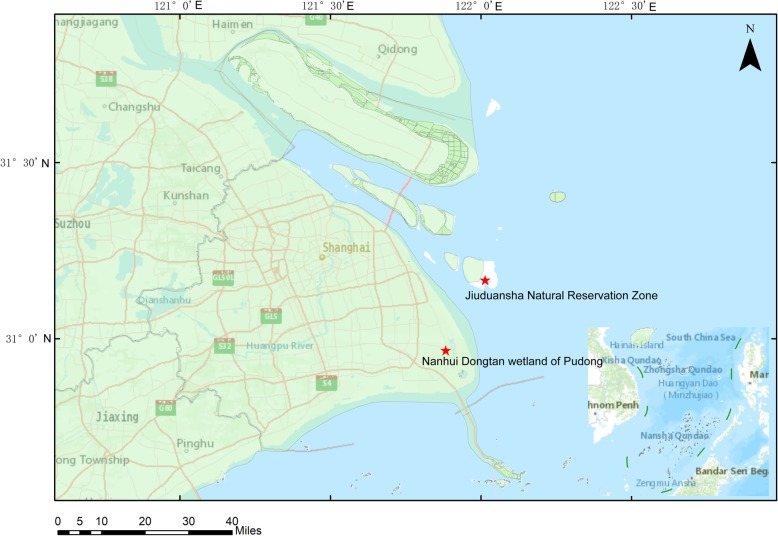
Map of two sampling sites at which the H6 subtype AIVs were isolated. The map was generated using ArcGIS version 10.4 (http://arcgis.com/)

We followed WHO guidelines for sample collection and preservation [15]. In spring and winter of 2016–2017, oropharyngeal and cloacal samples were collected from apparently healthy wild birds in Nanhui Dongtan wetland of Pudong and Jiuduansha Natural Reservation Zone, Shanghai, China (Fig. 1). These samples were collected using cotton swabs and placed into 5 ml Eppendorf (EP) tubes with 2 ml of viral transport media, and then frozen and stored at − 80 °C for further molecular analysis. The wild birds were captured and sampled with the permission and supervision of the Shanghai Wild Life Conservation and Management Office, and all birds were released after sampling.

### Virus identification and isolation

All experiments were conducted under biosafety level (BSL)-2 conditions. The swab samples were vortexed, oscillated, and centrifuged, and then the supernatants were collected. Virus RNAs were extracted directly from the collected supernatant using the MagMAX™ Pathogen RNA/DNA Kit (Applied Biosystems, USA) on a Magmax-96 Express instrument (Applied Biosystems) according to the manufacturer’s protocol. The positive samples of Influenza A viruses were confirmed using real-time PCR with the matrix gene primer and probe set [16]. Among these positive samples, the H6-positive samples were determined using real-time PCR with primers and probe set refered to a published paper [17]. Real-time PCR master mix was prepared according to the manufacturer’s instruction (One Step PrimeScriptTMRt-PCR Kit, TaKaRa, Japan). The partial sequences of HA gene and NA gene of all positive samples were amplified by ordinary PCR using universal primers based on WHO manual on animal influenza diagnosis and surveillance [18] and primers described in a previous report [19]. Then the partial sequences of HA and NA gene of all positive samples were compared with the sequences in GeneBank and the subtypes were confirmed. The positive controls used in this paper was H9N2 virus stored in our laboratory [20]. The selected H6-positive samples were inoculated into 9 to 10-day-old specific pathogen free (SPF) chicken embryos [18]. The inoculated chicken embryos were incubated for 72 h at 37 °C under humid conditions and then chilled at 4 °C for 6–8 h. The allantoic fluids from the inoculated chicken embryos were checked using a hemagglutination assay (HA) with 1% chicken red blood cells according to the manual above, too. Hemagglutinin-positive allantoic fluid was collected and stored at − 80 °C before use.

### RT-PCR and genome sequencing

Virus’ RNA was extracted from allantoic fluid using an Rneasy Mini kit (Qiagen, Hilden, Germany) and transcribed into cDNA using the Uni12 primer (5′-AGC AAA AGC AGG-3′) and PrimScript™ II 1st Strand cDNA Synthesis Kit (Takara, Japan). The eight segments of the H6 subtype AIVs were then amplified using the universal primers [19]. The PCR reaction contained 1 μL of cDNA, 1 μL of forward and reverse primers, 12.5 μL of Taq HS Perfect Mix (Takara) and 10.5 μL Rnase-free water, with a final volume of 25 μL. All sequences were confirmed using a BigDye termination kit (Applied Biosystems, Foster City, CA, USA) on an ABI 3730 sequence analyzer.

### Sequence analysis

For sequence data analysis and alignment, the DNAMAN program (version 6.0) was used. The phylogenetic trees were generated using the neighbor-joining algorithm and the 2-parameter model with bootstrap analysis (1000 replicates) in MEGA version 6 (http://www.megasoftware.net/). The other most relevant sequences to our isolates in the BLAST output were obtained from GenBank (https://www.ncbi.nlm.nih.gov/) and the Global Initiative on Sharing Avian Influenza Data (GISAID) EpiFlu database (https://platform.gisaid.org/epi3/frontend#2de575) for phylogenetic analysis.

### Pathogenicity to mice

To assess the pathogenicity of these wild bird-origin H6 AIVs in mice, 6–8-weeks-old BALB/c mice (Shanghai Jiesijie Experimental Animal Co., Ltd., China) were lightly anesthetized with ethylether and inoculated intranasally (i.n.) with 50 μL of 10^6^ EID50 of each virus, 8 mice in each group. The control mice were inoculated i.n. with an equal amount of noninfectious allantoic fluid. Three mice in each group were euthanized on day 3 p.i., the whole lungs were collected and weighed, and then were stored at − 80 °C for viral titration on MDCK cells. The pulmonary index was calculated according to the following formula: pulmonary index = [lung weight (g)/body weight (g)] × 100. The remaining 5 mice in each group were monitored daily for clinical symptoms, body weight, and survival time for a total of 14 days after virus infection.

### Statistical analysis

All data were analyzed using the Statistical Package for Social Science version 18.0 (SPSS, Inc., Chicago, IL), and results were expressed as the mean ± SEM. The one-way analysis of variance (ANOVA) followed by unpaired two-tailed t-test was used to evaluate the statistical significance of differences between two groups. *P*-values less than 0.05 were considered statistically significant.

## Results

### Isolation of H6 subtype avian influenza viruses from samples collected in Shanghai

Of 3290 cloacal and tracheal swab samples collected from apparently healthy wild birds in Shanghai, China, during 2016–2017, 271 samples (8.24%) were tested positive for influenza A virus, and 30 H6 samples were determined by real-time PCR. Of these H6 samples, 24 were detected in Common teal (*Anas crecca*), four were detected in Shoveller ducks (*Anas clypeata*), one was detected in Mandarin duck (*Aix galericulata*), and one was detected in Common Moorhen (*Gallinula chloropus*). Eight H6 positive strains were selected to perform further sequence analysis based on the subtypes, the cycle threshold (CT) values of real-time PCR and the homology of their partial HA sequences. There were 16 strains shared high nucleotide identity with the 8 strains selected in this study, while the quality and concentration of the remaining 6 strains (including one sample isolated from Common Moorhen) were too low to obtain the gene sequences (data not shown). The 8 strains consisted of four NA subtypes, H6N1 (A/Shoveller duck/Shanghai/PD1026–10/2016 and A/Shoveller duck/Shanghai/JDS1108–37/2017), H6N2 (A/Mandarin duck/Shanghai/PD1018–15/2017 and A/Common teal/Shanghai/PD1027–12/2017), H6N5 (A/Shoveller duck/Shanghai/PD1018–32/2017) and H6N8 (A/Common teal/Shanghai/PD1026–12/2016, A/Common teal/Shanghai/PD1026–19/2016 and A/Common teal/Shanghai/PD1109–24/2016) (Table 1).

**Table 1:** H6 subtype AIVs in this study

Virus	Abbreviation	Isolation date	Place
A/shoveller duck/Shanghai/PD1026–10/2016(H6N1)	PD1026–10(H6N1)	26-10-2016	Nanhui Dongtan wetland of Pudong
A/shoveller duck/Shanghai/JDS1108–37/2017(H6N1)	JDS1108–37(H6N1)	8-11-2017	Jiuduansha Natural Reservation Zone
A/mandarin duck/Shanghai/PD1018–15/2017(H6N2)	PD1018–15(H6N2)	18-10-2017	Nanhui Dongtan wetland of Pudong
A/commol teal/Shanghai/PD1027–12/2017(H6N2)	PD1027–12(H6N2)	27-10-2017	Nanhui Dongtan wetland of Pudong
A/shoveller duck/Shanghai/PD1018–32/2017(H6N5)	PD1018–32(H6N5)	18-10-2017	Nanhui Dongtan wetland of Pudong
A/commol teal/Shanghai/PD1026–12/2016(H6N8)	PD1026–12(H6N8)	26-10-2016	Nanhui Dongtan wetland of Pudong
A/commol teal/Shanghai/PD1026–19/2016(H6N8)	PD1026–19(H6N8)	26-10-2016	Nanhui Dongtan wetland of Pudong
A/commol teal/Shanghai/PD1109–24/2016(H6N8)	PD1109–24(H6N8)	9-11-2016	Nanhui Dongtan wetland of Pudong

### Phylogenetic analysis of H6 subtype avian influenza viruses

The whole genome sequences of these H6 subtype AIVs were analyzed, and pairwise alignment showed that nucleotide sequence identity among the eight gene segments shared a low similarity (83.8 to 99.9% for the HA gene, 49.2 to 99.9% for the NA gene, 90.9 to 100% for the M gene, 90.8 to 100% for the NP gene, 70.4 to 100% for the NS gene, 92.7 to 100% for the PB1 gene, 86.3 to 99.8% for the PB2 gene, and 88.3 to 100% for the PA gene), indicated that the eight H6 strains’ genomes were highly divergent. BLAST (https://www.ncbi.nlm.nih.gov/blast/) search results showed that except for the NS gene of A/common teal/Shanghai/PD1026–12/2016(H6N8), which exhibited the highest sequence identity with North American country-Alaska strain, the other gene segments of the eight isolates shared > 98.0% identity with those AIVs identified in East Asian countries (including Japan, Korea, Mongolia, and China), a South Asian country (Bangladesh) and a Southeast country (Vietnam) (Table 2), which are all located along the East Asian-Australasian flyway routes.

**Table 2:** Nucleotide identities of the highest homologs in GenBank database with the H6 subtype AIVs isolated in this study

Virus	Gene	Closest relative	Identity
A/shoveller duck/Shanghai/PD1026–10/2016(H6N1)	PB2	A/duck/Mongolia/777/2015(H3N8)	98.8%
	PB1	A/Duck/Dongting/D76–1/2016(H5N7)	99.4%
	PA	A/Duck/Dongting/D76–1/2016(H5N7)	99.5%
	HA	A/duck/Bangladesh/31227/2016(H6N2)	99.3%
	NP	A/Duck/Dongting/D76–1/2016(H5N7)	99.7%
	NA	A/wild bird/Wuhan/WHHN16/2014(H1N1)	99.3%
	M	A/duck/Bangladesh/31227/2016(H6N2)	99.8%
	NS	A/Pigeon/Longquan/LQ67/2016(H2N8)	99.3%
A/shoveller duck/Shanghai/JDS1108–37/2017(H6N1)	PB2	A/duck/Mongolia/520/2015(H1N1)	98.4%
	PB1	A/duck/Mongolia/520/2015(H1N1)	99.2%
	PA	A/duck/Fujian/SD013/2017(H6N6)	99.0%
	HA	A/duck/Ganzhou/GZ151/2016(H6N6)	98.8%
	NP	A/duck/Mongolia/709/2015(H10N7)	98.5%
	NA	A/wild bird/Wuhan/WHHN16/2014(H1N1)	98.0%
	M	A/duck/Guangdong/S4251/2010(H6N6)	98.7%
	NS	A/duck/Hubei/ZYSYG8/2015(H6N2)	98.3%
A/mandarin duck/Shanghai/PD1018–15/2017(H6N2)	PB2	A/duck/Vietnam/LBM798/2014(H3N6)	98.6%
	PB1	A/Duck/Dongting/D76–1/2016(H5N7)	99.3%
	PA	A/common porchard/Yamaguchi/3501B002/2017(H5N6)	98.5%
	HA	A/duck/Yamagata/061004/2014(H6N6)	98.3%
	NP	A/duck/Hokkaido/166/2014(H5N2)	99.0%
	NA	A/black-necked crane/Zhaotong/ZT-12/2013(H1N2)	98.7%
	M	A/duck/Hokkaido/W280/2014(H5N3)	98.8%
	NS	A/*Anser fabalis*/China/Anhui/S39/2014(H6N2)	99.0%
A/commol teal/Shanghai/PD1027–12/2017(H6N2)	PB2	A/duck/Vietnam/LBM798/2014(H3N6)	98.8%
	PB1	A/Duck/Dongting/D76–1/2016(H5N7)	99.2%
	PA	A/common porchard/Yamaguchi/3501B002/2017(H5N6)	98.3%
	HA	A/duck/Yamagata/061004/2014(H6N6)	98.1%
	NP	A/duck/Hokkaido/166/2014(H5N2)	99.0%
	NA	A/black-necked crane/Zhaotong/ZT-12/2013(H1N2)	98.6%
	M	A/duck/Hokkaido/W280/2014(H5N3)	98.8%
	NS	A/Anser fabalis/China/Anhui/S39/2014(H6N2)	99.0%
A/shoveller duck/Shanghai/PD1018–32/2017(H6N5)	PB2	A/duck/Mongolia/154/2015(H1N2)	98.8%
	PB1	A/Duck/Dongting/D76–1/2016(H5N7)	99.5%
	PA	A/common porchard/Yamaguchi/3501B002/2017(H5N6)	98.9%
	HA	A/duck/Bangladesh/31227/2016(H6N2)	99.1%
	NP	A/wildwater fowl/Hong Kong/MPL1006/2011(H7N7)	99.2%
	NA	A/migratory duck/Jiangxi/30246/2013(H10N5)	98.5%
	M	A/hooded crane/Korea/1176/2016(H1N1)	99.3%
	NS	A/duck/Jiangxi/22960/2008(H10N8)	99.4%
A/commol teal/Shanghai/PD1026–12/2016(H6N8)	PB2	A/duck/Hokkaido/103/2014(H3N8)	99.4%
	PB1	A/duck/Hokkaido/103/2014(H3N8)	98.8%
	PA	A/duck/Hokkaido/207/2014(H8N2)	99.2%
	HA	A/wild bird/Jiangxi/P419/2016(H6N8)	98.0%
	NP	A/duck/Hokkaido/103/2014(H3N8)	99.6%
	NA	A/duck/Hokkaido/91/2014(H3N8)	99.4%
	M	A/duck/Hokkaido/91/2014(H3N8)	100.0%
	NS	A/northern pintail/Alaska/496/2012(H3N8)	99.9%
A/commol teal/Shanghai/PD1026–19/2016(H6N8)	PB2	A/duck/Hokkaido/W9/2015(H1N1)	98.9%
	PB1	A/Duck/Dongting/D76–1/2016(H5N7)	99.5%
	PA	A/Duck/Dongting/D76–1/2016(H5N7)	99.3%
	HA	A/duck/Guangdong/G1345/2014(H6N6)	98.3%
	NP	A/duck/Ganzhou/GZ5/2015(H4N6)	99.2%
	NA	A/duck/Mongolia/487/2011(H3N8)	98.4%
	M	A/duck/Hubei/ZYSYF8/2015(H6N6)	98.8%
	NS	A/duck/Hubei/ZYSYG8/2015(H6N2)	99.3%
A/commol teal/Shanghai/PD1109–24/2016(H6N8)	PB2	A/duck/Hokkaido/W9/2015(H1N1)	98.9%
	PB1	A/Duck/Dongting/D76–1/2016(H5N7)	99.4%
	PA	A/Anseriformes/Anhui/L167/2014(H1N1)	98.8%
	HA	A/duck/Guangdong/G1345/2014(H6N6)	98.3%
	NP	A/duck/Ganzhou/GZ5/2015(H4N6)	99.3%
	NA	A/duck/Mongolia/487/2011(H3N8)	98.2%
	M	A/duck/Hubei/ZYSYF8/2015(H6N6)	98.6%
	NS	A/duck/Bangladesh/24706/2015(H7N1)	99.3%

**Fig. 2:**
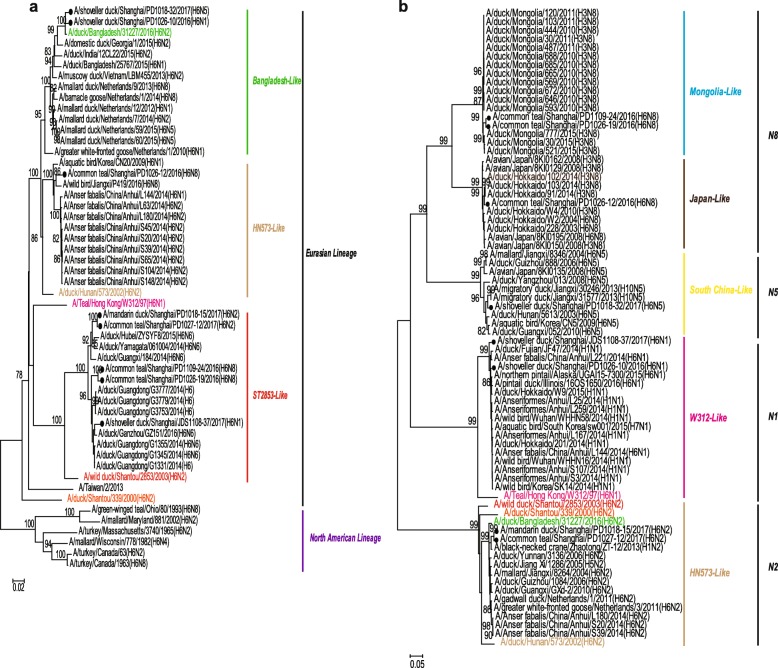
Phylogenetic tree of the HA (**a**) and NA (**b**) genes. The HA (**a**) and NA (**b**) genes of the H6 subtype AIVs from this study are indicated by black circles and the selected representative strains are shown in different colours. The neighbor-joining tree was constructed using the Kimura 2-parameter model in MEGA software version 6 (http://www.megasoftware net/). Bootstrap values were calculated on 1000 replicates, and values less than 75% are not shown. Numbers indicate neighbor-joining bootstrap values

Phylogenetic analysis of the HA genes suggested that the eight strains were all clustered into the Eurasian Lineage and could be further classified into three subgroups based on a previous study [7]. Five strains, PD1109–24(H6N8), PD1026–19(H6N8), PD1018–15(H6N2), PD1027–12(H6N2) and JDS1108–37(H6N1), grouped with the ST2853-like lineage represented by A/wild duck/Shantou/2853/2003(H6N2) (ST2853-like). One H6N8 strain, PD1026–12(H6N8), grouped with HN573-like lineage represented by A/duck/Hunan/573/2002(H6N2) (HN573-like). The other two strains PD1026–10(H6N1) and PD1018–32(H6N5), as a sister relationship with HN573-like virus, presented a low similarity (92.4–92.5%) with PD/1026–12(H6N8) and closely clustered with A/duck/Bangladesh/31227/2016(H6N2), as well as other strains coming from wild birds in Vietnam and Netherlands (Fig. 2a).

Phylogenetic analysis of the NA genes divided them into four groups: N1, N2, N5, and N8. Specifically, the two H6N8 strains, PD1109–24 (H6N8) and PD1026–19 (H6N8), clustered together and showed a close relationship with the NA gene of H3N8 viruses circulating in Mongolian ducks during 2010–2015, while another H6N8 strain grouped with the duck H3N8 viruses circulating in Japan in 2014. The two N2 isolates had a high similarity to the A/black-necked crane/Zhaotong/ZT-12/2013(H1N2) and clustered together in a small group. The strain PD1018–32(H6N5) was clustered with the H10N5 strain A/duck/Jiangxi/30246/2013(H10N5). The two N1 strains, PD/1026–10(H6N1) and JDS/1108–37(H6N1), were closely related to the wild bird H1N1 viruses circulating in China in 2014 and clustered with A/Teal/Hong Kong/W312/97(H6N1) that has the same seven genes with the strain H5N1 that could infect humans [9, 10] (Fig. 2b).

**Fig. 3:**
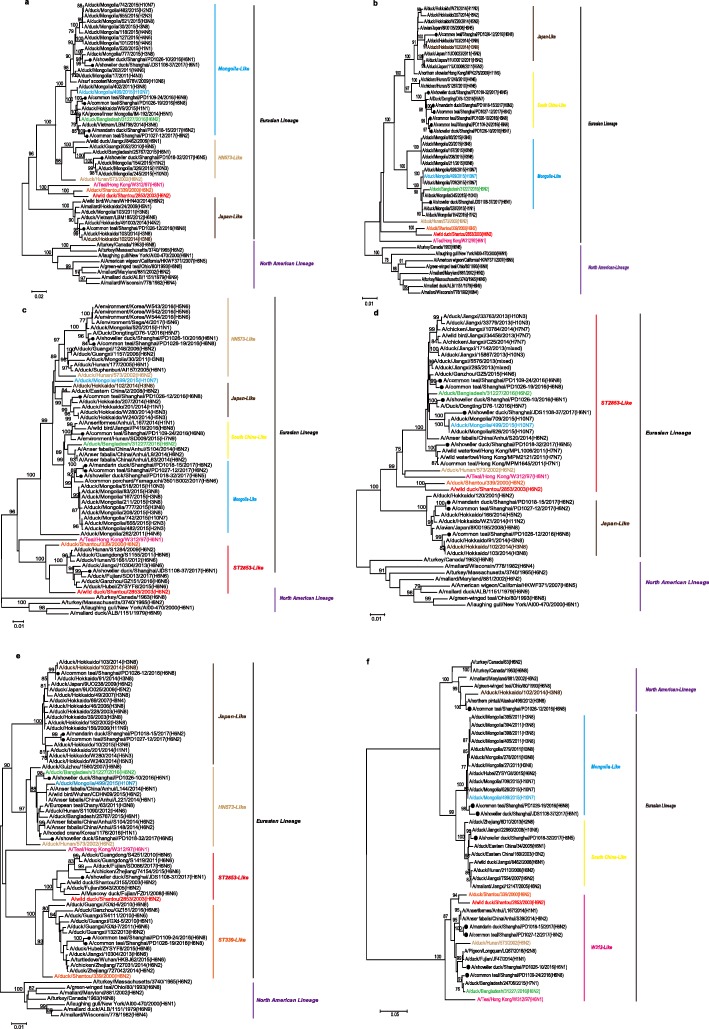
Phylogenetic tree of the PB2, PB1, PA, NP, M, and NS genes. The PB2 (**a**), PB1 (**b**), PA (**c**), NP (**d**), M (**e**), and NS (**f**) genes of the H6 subtype AIVs from this study are indicated by black circles and the selected representative strains are shown in different colours. The neighbor-joining tree was constructed as shown in Fig. 2

Phylogenetic analysis of the internal genes indicated that all eight H6 isolates belong to the Eurasian lineage, except the NS gene of the strain PD1026–12(H6N8), which was classified into the North American lineage. Phylogenetic analyses of the PB2 genes revealed the eight H6 strains belonged to three different groups in the Eurasian lineage, with strain PD1026–12(H6N8) clustering with the H3N8-like viruses circulating in Japan, strain PD1018–32(H6N5) clustering into the HN573-like sublineage, and the other six strains were most closely related to viruses from Mongolia (Fig. 3a). In the PB1 gene tree, except for strains PD1026–12(H6N8) and JDS1108–37(H6N1), which were closely related to H3N8 Japan-like or H10N3 Mongolia-like strains, respectively, the other six strains were clustered with the H5 subtype virus A/Duck/Dongting/D76–1/2016(H5N7) (Fig. 3b). Similarly, three of the PA genes had close relationships with those of a highly pathogenic H5N6 virus isolated from wild birds circulating in Japan in 2017. The other five PA genes were divided into four groups: One is closely related to H6N8 viruses from Jiangxi, one originated from Japan, and the other three grouped with HN573-like and ST2853-like lineages, respectively (Fig. 3c). For the NP genes, the eight strains were classified into two groups. Five strains, PD1026–10(H6N1), PD1109–24(H6N8), PD1026–19(H6N8), JDS1108–37(H6N1), and PD1018–32(H6N5), were grouped with the ST2853-like lineage, while the remaining three strains were closely clustered with strains from Japan (Fig. 3d). The M genes of all eight strains were placed into four groups: Three grouped with strains from Japan, two originated from the HN573-like lineage, one was from the ST2853-like lineage, and the remaining two originated from the ST339-like lineage (Fig. 3e). Furthermore, the NS phylogenetic tree showed that the eight H6 subtype AIVs were clustered into the North American lineage, Mongolia-like lineage, South China-like lineage, and the W312-like lineage (Fig. 3f). These results indicated that the internal genes of our strains exhibited great diversity and they all originated from multiple sublineages circulating in wild birds and domestic poultry along the migratory flyway.

**Fig. 4:**
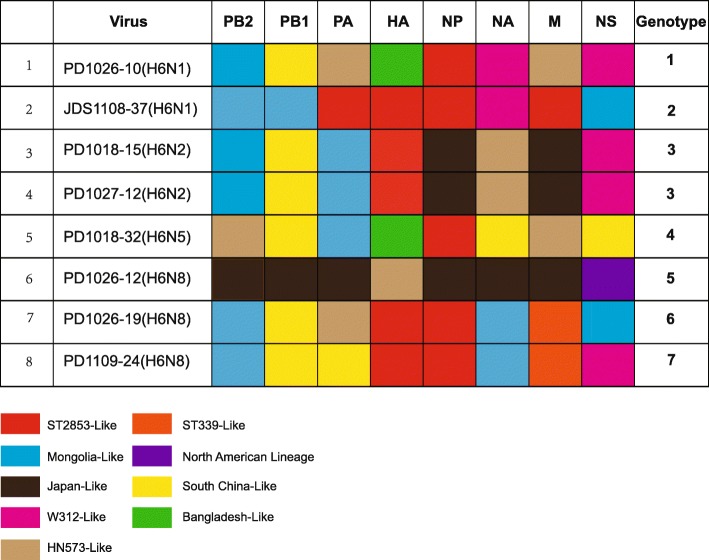
The genotypes of the eight H6 subtype AIVs. The eight H6 subtype AIVs were reassortants from 9 subgroups, including ST2853-like, HN573-like, Mongolia-like, W312-like, Bangladesh-like, Japan-like, ST339-like, South China-like and North American-lineage. According to different combinations, the eight viruses were divided into seven genotypes

Based on the topological structure of the eight full-length genome sequences of our isolates and the previous published papers [7, 21], these H6 genotypes were defined by two criteria: (i) phylogenetic analyses showed that these H6 sequences should belong to different sub-lineages (including ST2853-like, HN573-like, Mongolia-like, W312-like, Bangladesh-like, Japan-like, ST339-like, South China-like and North American-lineage), respectively, and it was supported by a bootstrap value above 70. (ii) the nucleotide identities of all sequences in the intergroup was less than 95%. According to these criteria, the 8 isolates in this study were assigned to 7 different genotypes (Fig. 4). The two H6N2 strains belonged to the same genotype, whereas the remaining six strains belonged to different genotypes. The intragroup homology of the two H6N2 strains was over 99.6%; they clustered together in a small subgroup and were reassorted with ST2853-like, Bangladesh-like, Japan-like, Mongolia-like, South China-like, and W312-like lineages. The two H6N1 strains belonged to two different genotypes, whose intragroup homology ranged from 70.6 to 98.9%, and were clustered into different sublineages, except for the NA, NP, and PB2 genes. The three H6N8 strains belonged to three different genotypes. The intragroup homology of PD1026–19(H6N8) and PD1109–24(H6N8) were greater than 99.4%, except for the NS and PA genes, but showed low similarity (70.8–95.2%) with another H6N8 strain, PD1026–12(H6N8). One H6N5 strain was reassorted from five sublineages, including a Bangladesh-like lineage, a South China-like lineage, a HN-573 like lineage, a ST2853-like, and the Mongolia-like lineage.

### Molecular characterization

All eight H6 subtype AIVs possessed a monobasic cleavage site (PQIETR↓GLF) between HA1 and HA2 (Table 3), indicating that these strains were characterized as low-pathogenic AIVs. The amino acids at the receptor binding sites, Q226 and G228 (H3 numbering), were highly conserved and no substitutions were found in the eight H6 subtype AIVs, showing that these viruses would preferentially bind to avian receptors (α-2, 3-galactose sialic acids, SA α-2, 3 Gal). Previous study [22] found H6 subtype AIVs had 6 potential N-linked glycosylation sites including four in HA1 (26 or 27, 39, 306 and 311) and two in HA2 (498 and 557). Our strains in this study had the same glycosylation sites in HA (data not shown). Except the strains PD1109–24 (H6N8) and PD1026–19 (H6N8) had an additional N-linked glycosylation site at position 67 to 69 on their NA proteins, the other common N-linked glycosylation sites in N1, N2, N5 and N8 also didn’t have any changes compared to published research [23]. Mutations involved in drug resistance (NA inhibitors and M2 inhibitors) were not observed in these strains, which suggested that these viruses maybe sensitive to neuraminidase inhibitors and M2 ion channel blockers. The substitutions in NS, PB1, or PB2 proteins that are associated with increased virulence to mammals were not found in the eight H6 strains.

**Table 3:** Molecular characteristics of H6 viruses in this study

Virus	Amino acidsequence at cleavage site of HA	Receptor-binding sites in HA (H3 numbers were used)				Key position in NA				M2	PB2		NS1
		138	186	190	226–228	119	275	293	295	31	627	701	92
1	PQIETR↓GLF	A	P	E	QRG	E	H	R	N	S	E	D	D
2	PQIETR↓GLF	A	P	E	QRG	E	H	R	N	S	E	D	D
3	PQIETR↓GLF	A	P	E	QRG	E	H	R	N	S	E	D	D
4	PQIETR↓GLF	A	P	E	QRG	E	H	R	N	S	E	D	D
5	PQIETR↓GLF	A	P	E	QRG	E	H	R	N	S	E	D	D
6	PQIETR↓GLF	A	P	E	QRG	E	H	R	N	S	E	D	D
7	PQIETR↓GLF	A	P	E	QRG	E	H	R	N	S	E	D	D
8	PQIETR GLF	A	P	E	QRG	E	H	R	N	S	E	D	D

### Pathogenicity of H6 viruses in mice

To investigate the pathogenicity of H6 viruses in mice, the maximum body weight loss, the pulmonary index and the virus titers in lungs were analyzed. Some mice in virus infected groups exhibited the obvious clinical features, such as inactivity, ruffled hair, and emaciation, but no deaths were observed. The maximum body weight loss in infected mice was observed on days 2–3 after H6 viruses infection, with the values ranging from 0.23 to 22.53 (Table 4). The lung index in virus-infected mice was increased compared with the control mice on 3 days post-infection, and the virus could be detected in lungs of all the infected mice. These results indicated that these H6 subtype influenza viruses could cause diseases in mice, and these viruses could replicate in the respiratory system without preadaptation, but couldn’t cause death.

**Table 4:** Pathogenicity of H6 influenza viruses in mice

Virus	Maximum body weight loss (%)	The pulmonary index on 3 days postinfection ^a^	Virus titers in lungs in mice (log_10_ TCID50/100 mg)
PD1026–10/2016(H6N1)	8.71	0.99 ± 0.37	4.08
PD1026–12/2016(H6N8)	10.95	0.88 ± 0.03	4.40
PD1026–19/2016(H6N8)	6.36	1.07 ± 0.17	2.74
PD1109–24/2016(H6N8)	0.23	0.80 ± 0.03	4.04
PD1018–15/2017(H6N2)	12.16	1.50 ± 0.40	7.85
PD1018–32/2017(H6N5)	17.80	1.45 ± 0.32	4.82
PD1027–12/2017(H6N2)	22.53	1.33 ± 0.09	4.19
JDS1108–37/2017(H6N1)	1.30	0.81 ± 0.03	3.44

^a^Data are presented as means ± standard deviations

## Discussion

Wild birds are generally accepted as a natural reservoir of avian influenza viruses [24]. Shanghai is located at East Asian-Australasian flyway route, and there are several critical stopover or wintering areas for migrating birds, such as Nanhui Dongtan wetland of Pudong and Jiuduansha Natural Reservation Zone. In this study, we collected samples from the above two important habitats for migratory birds in Shanghai, China, during 2016–2017, and the prevalence rate of the H6 viruses was 0.91% (30/3290), which is lower than the prevalence in poultry in eastern China (2.68%) [14], but higher than that in wild birds in southeastern China (0.44%) [21]. The different prevalence could be attributed to the different species and sample types. Among the influenza A virus positive samples in this study, about 11.07% of them were the H6 subtypes, indicating that H6 subtype might was one of the most frequently detected subtypes in migratory waterfowls in eastern China.

In this study, we found that the common teal was the major host of these H6 subtype AIVs, accounting for 80% of the total positive hosts. The common teal (*Anas crecca*) is one of the most abundant duck species in Shanghai, and usually undertakes fall migration from late October to March of the next year, and most of them winter in Shanghai (our unpublished research). Moreover, it is estimated that common teals in Eastern China account for 41% of the total number of that in Eastern Asia [25]; therefore, the common teal might play an important role in the ecology and epidemiology of influenza viruses in this area. In another surveillance along the East Asia-Australia migratory flyway, the host of all H6 viruses was bean goose (*Anser fabalis*) [21]. However, due to the less quantity and the difficulty of catching, we did not collected samples from bean goose in Shanghai.

The genetic results of the present study demonstrated that at least four H6 subtypes (H6N1, H6N2, H6N5, and H6N8) were co-circulating in wild birds in Shanghai. According to our knowledge, 10 H6N5 viruses have been isolated from domestic ducks in China in the database of Global Initiative on Sharing All Influenza Data (GISAID), but it has been not isolated from wild birds. Wild birds may provide conditions for the emergence of new subtype of AIVs. Previous studies showed that the H6N2 virus was one of the most frequently detected subtypes from 2002 to 2008 in domestic ducks in Eastern China, and from 2009 onwards, however, they were replaced by the novel H6N6 viruses [14]. The H6N2 virus also accounted for the majority of H6 AIVs (76.92%) in wild birds in southeastern China [21]. In this study, we did not detect the H6N6 viruses, the possible reasons were our limited number of samples or the H6N6 subtype were not common in wild birds in this region.

Phylogenetic analysis of these H6 subtype AIVs indicated that: 1) all of gene segments of the eight H6 strains clustered into the Eurasian lineage, except the NS gene of strain PD1026–12(H6N8), which belong to the North American lineage. Thus, gene flow might be present between the North American and the Eurasian lineages because of the overlapping routes of wild birds between Eurasia and the Americas. 2) The eight H6 strains in our study shared high sequence identity with those isolated from domestic ducks or wild birds in different countries along the East Asian-Australasian Flyway route, such as China, Japan, Korea, and Mongolia. The diversified gene exchange among different sublineages further confirmed that wild birds should play an important role in the spread of AIVs between wild birds and poultry [26]. 3) Interestingly, three PA genes of our isolates showed the highest nucleotide sequence identities with the H5N6 HPAIV (A/common porchard/Yamaguchi/3501B002/2017) in Japan. In 2014, HPAIV H5N6 virus from domestic ducks was first reported in Laos [27]. Subsequently, the epidemic of this subtype AIV in poultry and wild birds has aroused wide concern globally [28, 29]. Internal gene exchange between H5N6 and H6 subtype AIVs may exist between these two subtypes, thereby increasing the risk of spread of AIVs across species. 4) There were at least seven new H6 genotypes co-circulating in wild birds in Shanghai, however, they did not group with the previously described new reassortment H6 subtype AIVs isolated from wild birds or domestic poultry in eastern China in 2014 [21]. Peng et al. [30] performed a large surveillance in poultry in China during 2011–2016, 35 representative isolates were selected and classified into 31 genotypes. However, the further sequence analysis demonstrated that our strains in wild birds are distinguished from those isolated from poultry in China. This suggest that interspecies transmissions of the H6 subtype virus from domestic ducks to terrestrial poultry are not common.

Amino acids 226 and 228 in the HA protein are closely related to the receptor binding properties of AIVs. Generally, the amino acid combination Q226 and G228 are for the avian receptor, while the combination L226 and S228 are for the human receptor [31, 32]. In this study, the receptor-binding sites of the eight H6 strains were similar in all the H6 subtype AIVs examined, suggesting that they would preferentially bind to the avian cell surface receptors. In addition to the receptor binding sites, glycosylation also affects the receptor-binding preferences, and the replication and pathogenicity of avian influenza viruses [33–35]. However, the function of the additional glycosites in the two H6N8 strains needs to be further studied. Results of pathogenicity studies in mice indicated that these viruses could replicate efficiently in BALB/c mice lungs without prior adaption and caused diseases, which indicated that H6 subtype viruses might pose a threat to human health [13]. The mechanism of the virulence of these H6 viruses in mice need to be further studied, and continued surveillance of the H6 influenza viruses circulating in wild birds were needed.

## Conclusions

In conclusion, the study reported here identified 30 H6 subtype AIVs from 3290 samples. The 30 H6 subtype AIVs included four subtypes H6N1, H6N2, H6N5 and H6N8. From these strains, 8 representative strains were selected. Genetic analysis showed the isolates shared homology with different AIV sub-lineages isolated from domestic ducks or wild birds in different countries along the East Asian-Australasian flyways, and were classified into 7 new genotypes. What’s more, the strain PD1026–12(H6N8) also showed evidence of intercontinental gene exchange between Eurasian and North America. These viruses could replicate in the lungs of mice without preadaptation, but couldn’t cause death in mice. With the migration of wild birds and the increasing poultry trade, H6 subtype AIVs have been spreading widely in southern China and the biological characteristics are increasingly complex. Therefore, we should strengthen the surveillance and research about H6 subtype AIVs in wild birds in China.

## Data Availability

Complete genome sequences of the 8 strains in this study were deposited in the NCBI (https://www.ncbi.nlm.nih.gov/) under accession numbers MH352241–MH352304.

## Abbreviations

AIVs: Avian influenza viruses
MEGA: Molecular Evolutionary Genetics Analysis
GISAID: Global Initiative on Sharing Avian Influenza Data
WHO: World Health Organization
BSL: Biosafety level
SPF: specific pathogen free
CT: cycle threshold
